# Divergent periodic and aperiodic EEG signatures of Propofol versus sevoflurane anesthesia: a comparative neurophysiological study

**DOI:** 10.3389/fmed.2026.1794626

**Published:** 2026-04-28

**Authors:** Ping Shen, Miao Da, Zhongxia Shen

**Affiliations:** Huzhou Third Municipal Hospital, The Affiliated Hospital of Wenzhou Medical University, Huzhou, China

**Keywords:** anesthesia, aperiodic, components, electroencephalography, general, Propofol, sevoflurane

## Abstract

**Background:**

Current clinical anesthesia monitors often utilize drug-invariant indices that simplify cortical dynamics, potentially overlooking pharmacological nuances. While Propofol and Sevoflurane are both GABAergic, they may induce distinct neural states. This study aimed to identify the divergent periodic and aperiodic EEG signatures that distinguish these two regimens during the steady-state maintenance phase.

**Methods:**

A retrospective analysis was conducted using data from an open-access clinical database comprising 44 surgical patients (Propofol group, *n* = 27; Sevoflurane group, *n* = 17). EEG data were extracted during the pharmacological steady-state (20 min post-loss of consciousness to 10 min pre-end of surgery). Seventeen features, including relative band power, alpha peak frequency, and aperiodic components, were then derived. A multivariate statistical framework utilizing subject-independent cross-validation and SHapley Additive exPlanations (SHAP) analysis was implemented to identify and rank the most discriminatory biological markers.

**Results:**

The multivariate model achieved high discriminatory performance with a rigorous subject-level accuracy of 91.43%. Relative theta power, theta-to-alpha ratio, and alpha peak frequency were identified as the primary differentiators, occupying the top tiers of the SHAP importance ranking. Specifically, the Sevoflurane group exhibited a distinct elevation in theta-band prominence and a significant downward shift in alpha peak frequency (8.78 Hz vs. 10.88 Hz for Propofol). Furthermore, the aperiodic exponent emerged as a critical discriminatory feature, demonstrating a significantly steeper background spectral slope under Sevoflurane (2.37 vs. 2.07 for Propofol, *p* = 0.039). Conversely, alpha bandwidth (*p* = 0.263) and signal complexity measures (e.g., spectral entropy, *p* = 0.721) provided negligible discriminatory value.

**Conclusion:**

Propofol and Sevoflurane maintain unconsciousness via distinct neurophysiological regimes. The differentiation between these two agents is primarily driven by structural oscillatory shifts, specifically theta-band prominence and alpha peak deceleration, along with steepened aperiodic background dynamics, rather than periodic bandwidth or overall signal complexity. These findings underscore the distinct cortical modulation patterns of different GABAergic anesthetics and support the development of agent-specific, multidimensional monitoring protocols to enhance precision in individualized brain state assessment.

## Background

General anesthesia is a reversible drug-induced state encompassing unconsciousness, amnesia, and analgesia. While foundational systems neuroscience has elucidated the broad mechanisms of arousal states during the previous decade (1, 2), the precise neurophysiological signatures that differentiate the cortical effects of diverse anesthetic regimens remain a subject of active contemporary investigation (3–5). In modern clinical practice, the assessment of anesthetic depth typically integrates traditional clinical indicators with processed electroencephalogram (EEG) indices, which serve as objective adjunctive tools (6, 7). Although these automated metrics provide a standardized reference point, recent high-level evidence, such as the eMODIPOD randomized controlled trial, indicates that a monolithic algorithmic approach may provide limited resolution of the nuanced neural dynamics associated with diverse pharmacological agents, particularly in the context of multimodal or opioid-free anesthesia (3, 7, 8). This clinical gap is relevant to individualized patient care, as a deeper understanding of drug-specific cortical dynamics, incorporating the integration of both periodic oscillations and aperiodic background activity, is intended to support the optimization of perioperative management and the mitigation of postoperative neurocognitive complications (5, 9, 10).

The neurophysiological signatures of the two most ubiquitous GABAergic agents, Propofol and Sevoflurane, demonstrate significant periodic (oscillatory) divergence that reflects their distinct modulatory effects on neural circuits. Propofol-maintained anesthesia is characterized by the emergence of highly stable, hypersynchronous alpha oscillations (8–13 Hz), which are thought to result from the enhancement of GABA_A_ receptor-mediated inhibitory postsynaptic currents within the thalamocortical feedback loops (11). In contrast, sevoflurane-maintained regimens, while also facilitating GABA_A_ pathways, frequently induce a distinct frontal theta-band prominence and a characteristic downward shift of the alpha peak frequency into the slow-alpha range (12–14). This rhythmic differentiation is not merely a spectral phenomenon but indicates a fundamental variation in how intravenous and volatile agents decouple cortical communication and disrupt information integration. Beyond these classical rhythmic variations, recent advancements in computational neuroscience and spectral decomposition have emphasized the critical importance of the aperiodic (non-oscillatory) component of the EEG power spectrum (5, 15). As highlighted in recent expert commentary, analyzing this background activity is no longer optional but essential for deciphering neural states under anesthesia (16). The aperiodic exponent, which characterizes the rate of power decay across the frequency spectrum, serves as a robust proxy for the global excitation-inhibition (E/I) balance of the cerebral cortex (5, 15, 17). A steeper exponent is associated with increased inhibitory conductance and decreased cortical dynamical stability, yet the specific divergence in these aperiodic signatures between different GABAergic agents remains insufficiently characterized, particularly during the steady-state maintenance phase of clinical anesthesia.

The identification of drug-specific neurophysiological markers is relevant to clinical practice, as deviations in spectral dynamics are associated with perioperative neurological outcomes. For instance, a reduction in alpha-band power and an increase in low-frequency oscillations are recognized as electrophysiological indicators of neurocognitive vulnerability and are linked to an increased risk of postoperative delirium and delayed recovery (18–20). In surgical populations with diminished neural reserve, the characterization of these signatures may facilitate the differentiation between intrinsic neurocognitive vulnerability and expected pharmacological effects. This differentiation represents a factor in preventing accidental over-sedation, especially considering the age-related paradoxes where commercial monitoring indices may fail to accurately reflect the hypnotic state in older patients (20–22).

While previous research established the feasibility of utilizing multivariate models to classify general states of unconsciousness under GABAergic anesthesia (23), the specific ranking of divergent periodic and aperiodic signatures that distinguish Propofol from sevoflurane during the pharmacological steady-state has not been fully established. By utilizing a multivariate statistical framework integrated with spectral decomposition, the current research aims to provide a neurophysiological basis for agent-specific brain state monitoring. This approach is intended to support the transition toward individualized perioperative care and the optimization of anesthetic titration to improve neurocognitive outcomes.

## Methods

### Data source and EEG preprocessing

The EEG data utilized in this study were obtained from the GABAergic Anesthetic-Induced Unconsciousness and Recovery database on PhysioNet (23). The analysis focused on a surgical cohort of 44 patients receiving clinical anesthesia. Patients were categorized into two groups based on the primary maintenance agent: the Propofol group (total intravenous anesthesia) and the Sevoflurane group (sevoflurane-based maintenance). Frontal EEG data were recorded using the Sedline brain function monitor (Masimo Corporation, Irvine, CA, United States). The standard Sedline Sedtrace electrode array recorded from electrodes located approximately at positions Fp1, Fp2, F7, and F8, with ground at Fpz and reference electrode approximately 1 cm above Fpz. The EEG data were acquired with a pre-amplifier bandwidth of 0.5–92 Hz, a sampling rate of 250 Hz, and 16-bit resolution (29 nV). Electrode impedance was maintained below 5 kΩ for each channel.

To ensure a robust comparison under pharmacological steady-state and exclude dynamic transitions during induction and emergence (24), EEG segments were extracted from the period starting 20 min after the loss of consciousness (LOC) until 10 min before the end of surgery.

EEG signals were segmented into non-overlapping 30-s epochs to optimize spectral resolution and ensure stable estimation of the aperiodic component, commonly referred to as 1/f activity, which represents the background power distribution of the spectrum (15, 25). A multi-stage automated artifact rejection pipeline was implemented to maintain signal integrity. First, signal dropouts and lead-off events were detected by identifying epochs with a total power significantly lower than the individual session mean. Second, broadband high-frequency interference (e.g., electrocautery) was excluded by calculating the total power in the 0.5–45 Hz range. Epochs exceeding 2.5 standard deviations (*μ* + 2.5σ) from the individual session mean were considered contaminated and discarded (20).

As detailed in Table 1, the cohort demographics exhibited a significant age difference (mean 50 years in the Sevoflurane group compared to 32 years in the Propofol group, *p* < 0.001) and divergent analysis durations per patient (*p* < 0.001). After the artifact rejection protocol, 113 epochs were excluded, resulting in a final analytical pool of 3,185 high-quality epochs, with 946 segments in the Propofol group and 2,239 in the Sevoflurane group. The artifact rejection rate was equivalent between the two regimens (3.47% vs. 3.41%, *p* = 0.932). To address the potential confounding effects of age-related absolute amplitude attenuation and the non-equivalence of monitoring indices across different ages, the feature selection strategy prioritized relative spectral power and frequency-based characteristics. This approach was implemented because relative and peak frequency metrics have been established as robust pharmacological signatures that maintain drug-specificity throughout the adult lifespan despite physiological changes associated with brain aging (21, 22, 26).

**Table 1:** Demographic characteristics and EEG data distribution.

Characteristics	Propofol group (*n* = 27)	Sevoflurane group (*n* = 17)	*p*-value
Age (years)	32 (range 18–59)	50 (range 18–90)	<0.001
Analysis duration per patient (min)	28.06 ± 4.2 min	66.01 ± 63.54 min	<0.001
Total clean epochs (N)	946	2,239	–
Artifact rejection rate (%)	3.47% (34/980)	3.41% (79/2318)	0.932

Age data are cited from the original study (23). Continuous variables are expressed as mean ± SD or range.

### Feature extraction and neurophysiological rationale

To characterize the maintenance phase of anesthesia, 18 neurophysiologically relevant features were extracted from each 30-s EEG epoch. These features were categorized into four functional domains: Relative Band Power, Spectral Ratios, Spectral Distribution and Oscillatory Rhythms, and Aperiodic and Complexity Components (Table 2).

**Table 2:** Definition and neurophysiological significance of extracted EEG features.

Category	Feature name	Mathematical/frequency definition
Relative power	Delta_Rel, Theta_Rel, Alpha_Rel, Beta_Rel, Gamma_Rel	Power in specific band / Total Power (0.5–30 Hz)
Spectral ratios	e.g., Theta_Alpha_Ratio	Ratio between power of specified bands
Oscillatory and distribution	Peak_Freq, Alpha_Bandwidth, SEF95, SEF50	Alpha peak properties and power distribution boundaries (95th & 50th percentile)
Aperiodic components	Exponent (Slope), Offset	Parameters from 1/f background model: $P(f)=L+f^{-x}$
Complexity	SpecEn	Shannon entropy of the normalized power spectrum

Relative Band Power: Relative power for five canonical frequency bands was calculated as the ratio of power within a specific frequency range to the total power of the analyzed spectrum (0.5–30 Hz). The extracted features included Delta_Rel (0.5–4 Hz), Theta_Rel (4–8 Hz), Alpha_Rel (8–13 Hz), Beta_Rel (13–25 Hz), and Gamma_Rel (25–30 Hz). The utilization of relative power metrics, rather than absolute amplitude, was implemented to minimize confounding factors associated with inter-individual variability in skull impedance and age-related power attenuation (22, 27).

Spectral Ratios: Five distinct power ratios were computed to capture the dynamic balance between different oscillatory circuits: Theta/Alpha (4–8 Hz/8–13 Hz), Theta/Delta (4–8 Hz/0.5–4 Hz), Beta/Delta (13–25 Hz/0.5–4 Hz), Beta/Alpha (13–25 Hz/8–13 Hz), and Alpha/Delta (8–13 Hz/0.5–4 Hz). These ratios quantify the competitive interaction between slow-wave activity and spindle-like oscillations (defined as waxing and waning rhythms within the 8–13 Hz alpha band) under GABAergic modulation (5, 6, 13).

Spectral Distribution and Oscillatory Rhythms: The Alpha Peak Frequency (Peak_Freq), defined as the frequency within the 8–13 Hz band exhibiting maximum power, and the Alpha Bandwidth were extracted as indices of thalamocortical loop integrity (11, 28). Additionally, the 95% spectral edge frequency (SEF95) and the median frequency (SEF50) were calculated to summarize the macroscopic distribution of spectral energy and monitor the degree of drug-induced cortical depression (29–31).

Aperiodic and Complexity Components: Decomposition of the power spectrum into periodic and aperiodic components was performed using the SpecParam (FOOOF) algorithm (5, 15). The Aperiodic Exponent (representing the 1/f slope) and Offset (broadband intercept) were extracted to index global excitation-inhibition (E/I) balance (15, 17). A steeper aperiodic slope (higher exponent) is associated with enhanced inhibitory tonic activity (17, 32). Furthermore, Spectral Entropy (SpecEn) was utilized as a non-linear measure to quantify the irregularity and information complexity of the spectral distribution (33, 34).

### Multivariate statistical framework and interpretability analysis

To identify the distinctive neurophysiological signatures of Propofol and Sevoflurane anesthesia, a multivariate statistical framework based on the Random Forest (RF) algorithm was implemented. The RF model was selected for its robust performance in handling high-dimensional, non-linear EEG features and its inherent resistance to overfitting in clinical datasets (35–37).

The classification task was conducted at the window level using 30-s epochs. To strictly prevent data leakage and ensure generalizability to new subjects, a subject-level 5-fold cross-validation scheme (GroupKFold) was implemented. This method safeguards against overfitting by ensuring that all epochs from a given patient are assigned exclusively to either the training or the testing set within any fold, thereby requiring the model to identify drug-specific signatures that transcend individual variability.

To prevent performance inflation caused by epoch-level autocorrelation, the final model evaluation was strictly conducted at the subject level. Following the subject-independent 5-fold cross-validation, the model generated predicted probabilities for all unseen 30-s epochs belonging to a given patient. These epoch-level probabilities were then arithmetically averaged to yield a composite subject-level probability score. The final classification for each patient was determined using a standard 0.5 threshold on this composite score. Model performance was primarily evaluated using subject-level accuracy, and a subject-level confusion matrix was generated to detail the classification sensitivity and specificity. To transition from a black-box predictive model to an interpretable neurophysiological framework, SHapley Additive exPlanations (SHAP) analysis was employed (37, 38). By calculating the mean absolute SHAP values, a feature importance ranking was established to identify the primary biological markers driving the differentiation of GABAergic agents.

### Statistical analysis

Continuous variables are presented as mean ± standard deviation (SD). To evaluate the neurophysiological divergence between the Propofol and Sevoflurane regimens, statistical comparisons for each EEG feature were performed using Welch’s *t*-test. This parametric approach was selected to account for the unequal sample sizes and potential heteroscedasticity (unequal variances) between the study cohorts. Statistical significance was defined as a two-tailed *p < 0.05*. All *p*-values are reported to three decimal places, with values less than 0.001 designated as <0.001. The Ranking of features was determined by the magnitude of the calculated *p*-values. All statistical analyses were conducted using Python 3.10 with SciPy 1.15.3.

## Results

### Divergence in periodic and aperiodic spectral profiles

**Figure 1:**
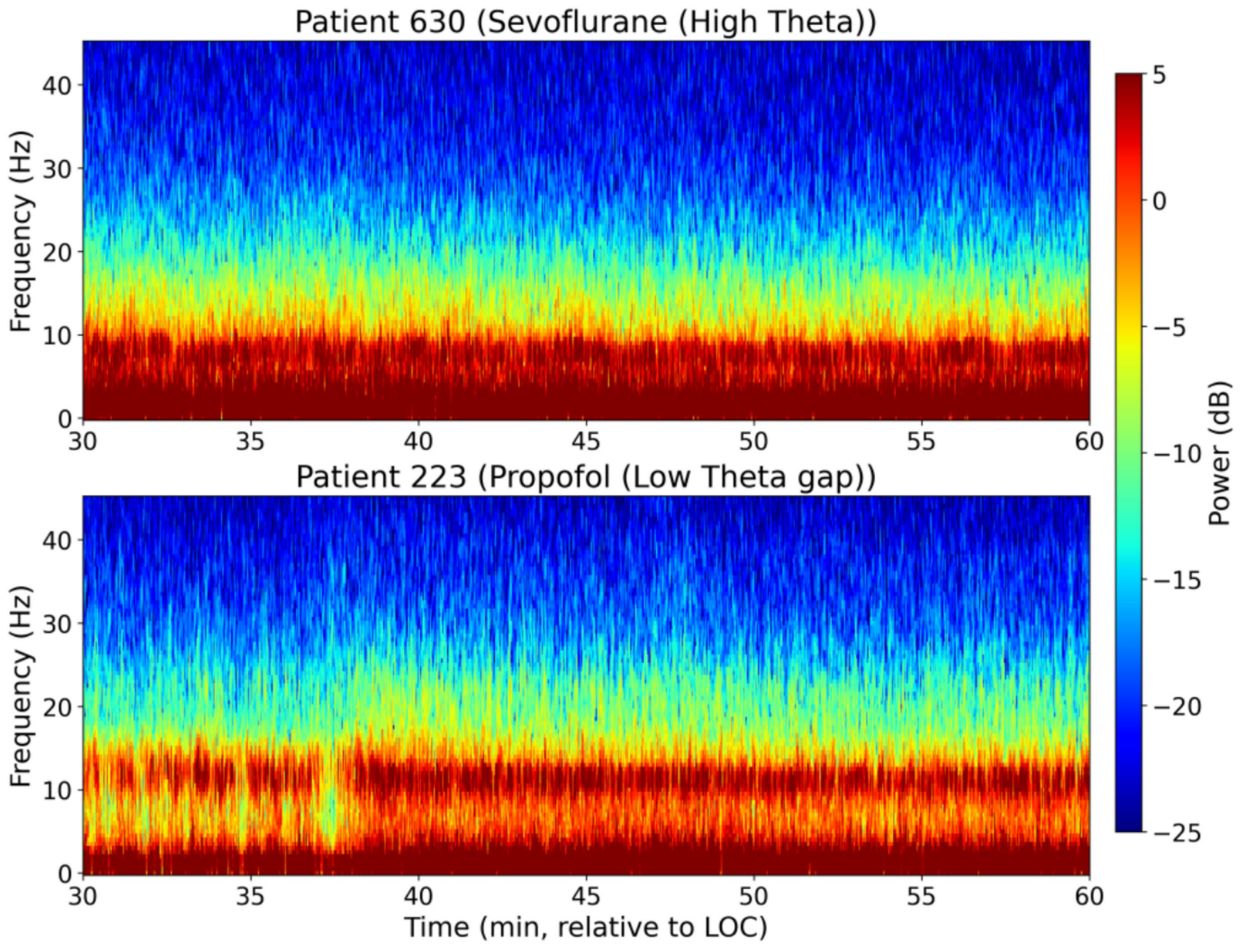
Representative frontal EEG spectrograms during maintenance anesthesia. Time-frequency dynamics are shown for a representative patient in the sevoflurane group (top) and the propofol group (bottom) during the steady-state maintenance period (30–60 min post-LOC). The sevoflurane group demonstrates a characteristic high-power band extending into the theta range, whereas the propofol group maintains a highly stable and rhythmic alpha oscillation.

Visual inspection of the representative spectrograms (Figure 1) during the steady-state maintenance phase revealed marked morphological differences between the two regimens. The Sevoflurane-maintained group was characterized by a broad-band power distribution with a prominent and persistent power elevation in the theta range (4–8 Hz). In contrast, the Propofol-maintained group exhibited a highly rhythmic and concentrated alpha-band oscillation with minimal spectral encroachment from adjacent frequency bands.

**Figure 2:**
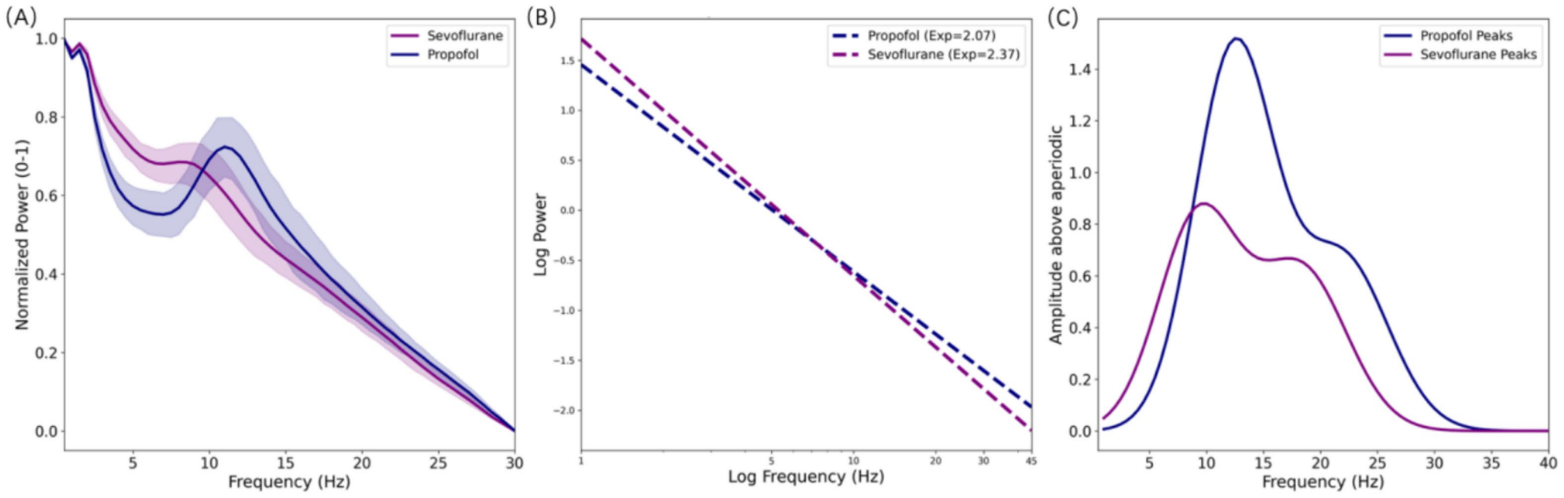
Spectral decomposition and aperiodic component analysis. **(A)** Normalized group power spectral density (PSD) highlighting the alpha peak shift between groups. **(B)** Aperiodic (1/*f*) background fitting on a log–log scale. The diverging slopes illustrate a significantly steeper decay exponent in the sevoflurane group (2.37) compared to the propofol group (2.07; *p* = 0.039), demonstrating that the distinct pharmacological modulation of volatile anesthesia overrides the expected age-related spectral flattening. **(C)** Flattened spectra showing isolated rhythmic peaks after subtracting the aperiodic background, further clarifying the divergence in dominant oscillation frequencies.

These qualitative observations were further substantiated through normalized group power spectral density (PSD) analysis (Figure 2A). The Propofol group demonstrated a sharp, high-amplitude alpha peak centered at approximately 11 Hz. Conversely, the Sevoflurane group exhibited a significant leftward shift in the dominant oscillatory frequency, with the peak residing in the slow-alpha range (~8.78 Hz). To distinguish between rhythmic oscillations and background dynamics, the power spectrum was decomposed into periodic and aperiodic components. The aperiodic fitting (Figure 2B) revealed a significant divergence in background spectral dynamics on a log–log scale: the Sevoflurane group exhibited a steeper aperiodic exponent compared to the Propofol group (2.37 ± 0.21 vs. 2.07 ± 0.40, *p* = 0.039; SHAP Rank 5). Physiologically, advancing age is strongly associated with a flattening of the power spectrum (decreased exponent). The observation that the significantly older Sevoflurane cohort (mean 50 years) not only maintained but exhibited a significantly steeper decay than the younger Propofol cohort (mean 32 years) indicates a profound pharmacological modulation. This robust drug-specific steepening effect of volatile anesthesia effectively overrides the expected age-related spectral flattening. Following the subtraction of the aperiodic background, the flattened spectra (Figure 2C) confirmed that the downward shift in dominant peak frequency and the broadened theta-band prominence in the Sevoflurane group were intrinsic oscillatory signatures, rather than spectral artifacts driven by the steeper 1/f decay.

### Robust classification and topological separability of drug signatures

**Figure 3:**
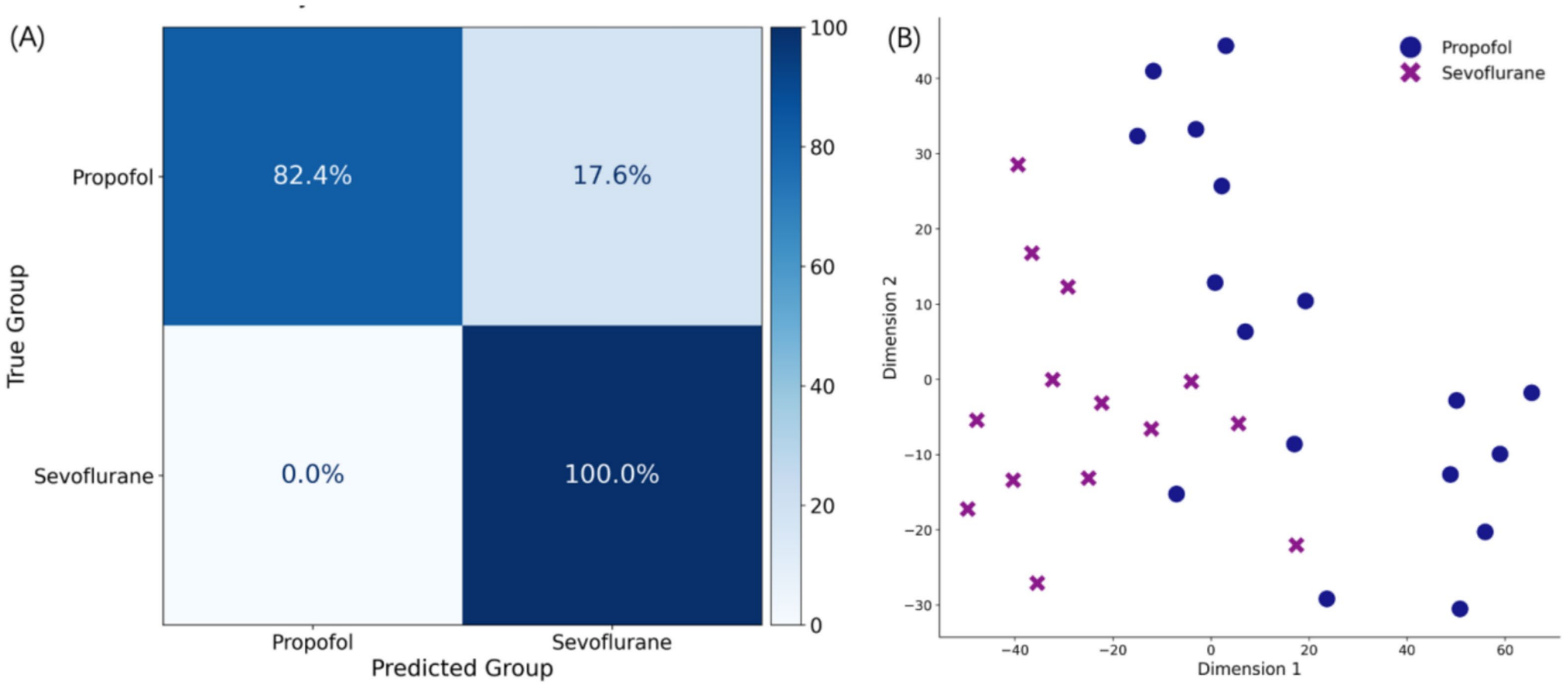
Subject-level multivariate classification performance. **(A)** The corresponding subject-level confusion matrix detailing the true positive and false positive classification rates across the two anesthetic regimens. **(B)** *t*-SNE visualization illustrating the spatial separability of propofol-induced (blue circles) and sevoflurane-induced (purple crosses) cortical states based on the 17-dimensional neurophysiological feature set. Each point represents an individual 30 s EEG epoch.

The multivariate statistical framework demonstrated a robust capacity to distinguish between Propofol and Sevoflurane anesthetic states. By aggregating epoch-level predictions to classify entirely unseen patients, the Random Forest classifier yielded a rigorous subject-level accuracy of 91.43%. This metric accurately reflects the model’s true clinical generalizability while preventing performance inflation from highly autocorrelated data. Furthermore, the corresponding subject-level confusion matrix (Figure 3A) confirmed the model’s sensitivity and specificity, correctly identifying the primary maintenance agent for the vast majority of individual patients. This high degree of classification accuracy indicates that the neurophysiological signatures associated with each GABAergic agent are profoundly distinctive and identifiable across individuals, effectively overriding intrinsic differences in demographic profiles.

To evaluate the topological structure of these signatures, t-Distributed Stochastic Neighbor Embedding (t-SNE) was utilized to project the feature set onto a two-dimensional manifold (Figure 3B). The t-SNE projection revealed distinct clustering of Propofol-induced and Sevoflurane-induced cortical states with minimal overlap. This spatial separability indicates that these agents transition the cortex into different dynamical regimes. The consistency of this classification under subject-independent testing suggests that drug-specific signatures override individual variability during the maintenance period.

### Feature importance and independence of discriminatory markers

**Figure 4:**
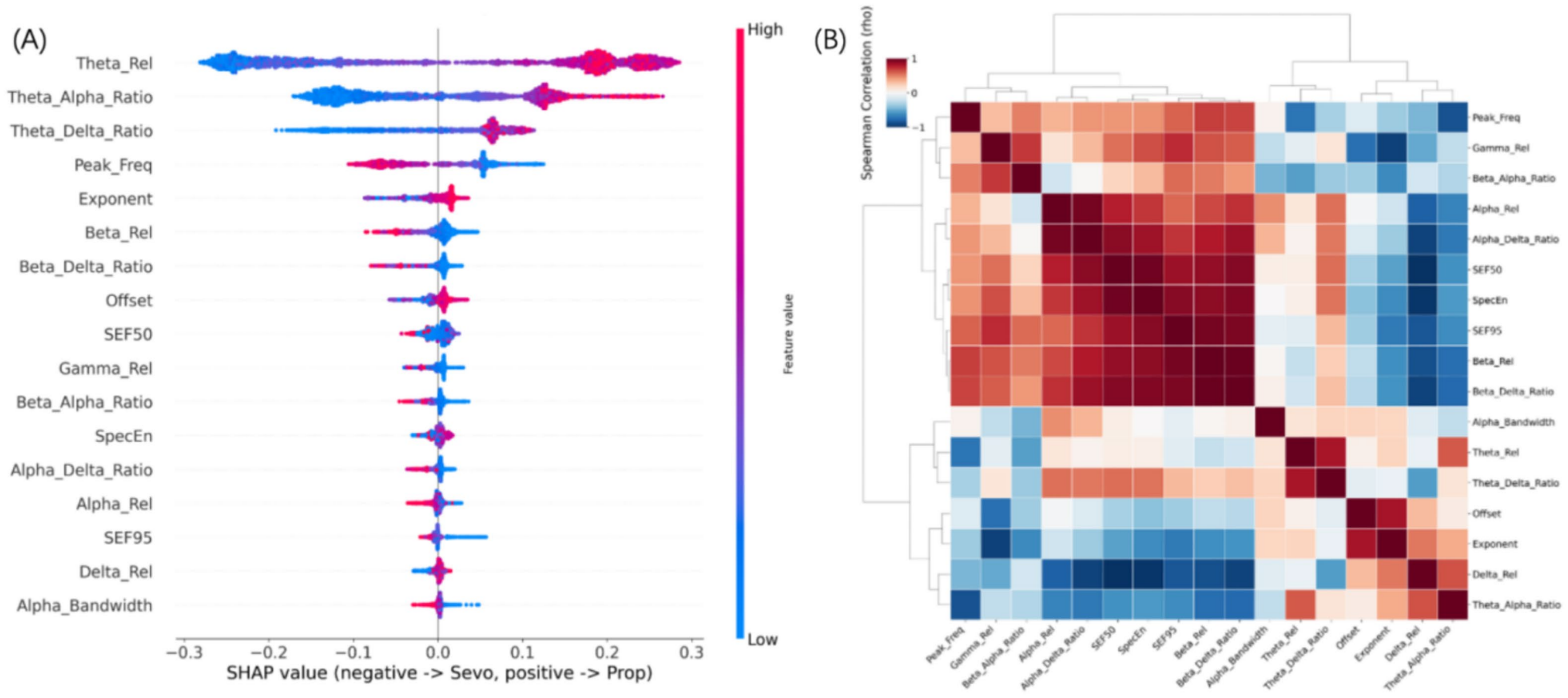
Multivariate feature importance and functional independence of discriminatory neurophysiological markers. **(A)** SHAP summary plot ranking the contribution of all 17 features to the classification model. Features are ordered by their mean absolute SHAP value. Each dot represents a 30 s epoch, with color indicating the original feature magnitude (pink: high, blue: low). Positive SHAP values drive the prediction toward Propofol, while negative values drive it toward Sevoflurane. The analysis identifies relative theta power, theta-to-alpha ratio, and alpha peak frequency as the primary drivers. **(B)** Spearman correlation heatmap with hierarchical clustering of the extracted features. The color scale represents the correlation coefficient ranging from −1 (blue) to +1 (red). The dendrograms reveal that the top-ranked predictors identified by SHAP (e.g., Theta_Rel, Peak_Freq, and Exponent) originate from distinct, largely independent functional clusters.

The relative contribution of each neurophysiological parameter to the identification of the anesthetic agent was elucidated using SHAP analysis (Figure 4A). The importance ranking revealed a profound dominance of theta-band dynamics, identifying Theta_Rel (Rank 1), Theta_Alpha_Ratio (Rank 2), and Theta_Delta_Ratio (Rank 3) as the absolute primary differentiators. These were immediately followed by two critical structural features of the power spectrum: the oscillatory Peak_Freq (Rank 4) and the aperiodic Exponent (Rank 5). Notably, Alpha_Bandwidth and signal complexity measures (e.g., SpecEn) provided negligible discriminatory value, ranking at the very bottom of the model. This confirms that agent differentiation relies on specific frequency shifts and aperiodic background steepness, rather than the width of periodic peaks or overall signal stochasticity.

The SHAP summary plot demonstrated consistent directional relationships linking specific neurophysiological states to each regimen. Specifically, elevated theta-band activity and higher theta-related power ratios were strongly associated with classification into the Sevoflurane group (driving negative SHAP values). Furthermore, a higher aperiodic exponent (a steeper spectral decay) also served as a critical predictor for Sevoflurane, underscoring its unique pharmacological modulation of background cortical dynamics. Conversely, a higher alpha peak frequency and increased relative beta power (Beta_Rel, Rank 6) served as hallmark signatures predictive of Propofol anesthesia (driving positive SHAP values). The heavy reliance on relative power, discrete peak frequencies, and aperiodic slope—rather than absolute amplitudes—underscores the model’s robustness against inter-individual variability and potential demographic confounders.

To assess potential redundancy and synergistic relationships among the 17 extracted features, a Spearman correlation matrix with hierarchical clustering was constructed (Figure 4B). The dendrograms revealed three functionally distinct clusters. A large, highly correlated block emerged in the upper left, predominantly capturing high-frequency oscillatory dynamics (e.g., Beta_Rel, Beta_Alpha_Ratio) and specific alpha characteristics (Peak_Freq). A second distinct block strictly captured theta-band power and its ratios (Theta_Rel, Theta_Delta_Ratio). Crucially, the aperiodic components (Exponent and Offset) clustered tightly together in the bottom right, exhibiting very weak or even negative correlations with the traditional periodic features. The fact that the absolute top-ranked predictors identified by the SHAP analysis (Theta_Rel, Peak_Freq, and the aperiodic Exponent) originate from these entirely independent clusters confirms a key methodological strength: the model’s superior classification performance stems from integrating complementary, non-redundant information streams spanning discrete slow-wave oscillations, specific peak frequency shifts, and global background decay.

### Comparative statistical distribution of key periodic and aperiodic biomarkers

A comprehensive summary of all 17 extracted features, including group means, SHAP importance rankings, and statistical comparisons, is provided in Table 3. The analysis revealed a robust divergence in periodic and oscillatory markers. Specifically, Theta_Rel, Theta_Alpha_Ratio, Theta_Delta_Ratio, Peak_Freq, and Exponent demonstrated significant group differences and consistently occupied the top tiers of the SHAP importance ranking.

**Table 3:** Statistical comparison of EEG features between Propofol and Sevoflurane groups.

Category	Feature	Propofol (*n* = 27)	Sevoflurane (*n* = 17)	Rank	*P*-value
Periodic (relative power)	Theta_Rel	[0.10 ± 0.03]	[0.20 ± 0.05]	1	**<0.001**
	Beta_Rel	[0.13 ± 0.12]	[0.06 ± 0.03]	6	**0.028**
	Gamma_Rel	[0.01 ± 0.02]	[0.00 ± 0.00]	10	0.116
	Delta_Rel	[0.60 ± 0.22]	[0.63 ± 0.12]	16	0.520
	Alpha_Rel	[0.17 ± 0.09]	[0.15 ± 0.07]	14	0.527
Periodic (ratios)	Theta_Alpha_Ratio	[0.65 ± 0.23]	[1.65 ± 0.84]	2	**0.001**
	Theta_Delta_Ratio	[0.22 ± 0.19]	[0.34 ± 0.13]	3	**0.028**
	Beta_Delta_Ratio	[0.42 ± 0.65]	[0.11 ± 0.08]	7	0.056
	Beta_Alpha_Ratio	[0.75 ± 0.52]	[0.42 ± 0.18]	11	**0.039**
	Alpha_Delta_Ratio	[0.45 ± 0.49]	[0.28 ± 0.23]	13	0.369
Oscillatory and distribution	Peak_Freq (Hz)	[10.88 ± 0.74]	[8.78 ± 0.87]	4	**<0.001**
	SEF95 (Hz)	[16.53 ± 6.01]	[13.05 ± 2.92]	15	0.068
	Alpha_Bandwidth	[4.73 ± 0.99]	[4.72 ± 1.17]	17	0.263
	SEF50 (Hz)	[3.91 ± 3.64]	[2.74 ± 1.30]	9	0.214
Aperiodic (1/f)	Exponent	[2.07 ± 0.40]	[2.37 ± 0.21]	5	**0.039**
	Offset	[1.46 ± 0.52]	[1.71 ± 0.34]	8	0.527
Complexity	SpecEn	[3.12 ± 0.52]	[3.11 ± 0.26]	12	0.721

Features with *p* < 0.05 are indicated in bold. *P*-values were calculated using Welch’s *t*-test and adjusted for multiple comparisons using the Benjamini-Hochberg procedure (FDR < 0.05). *p*-values are reported to three decimal places; values less than 0.001 are designated as <0.001.

**Figure 5:**
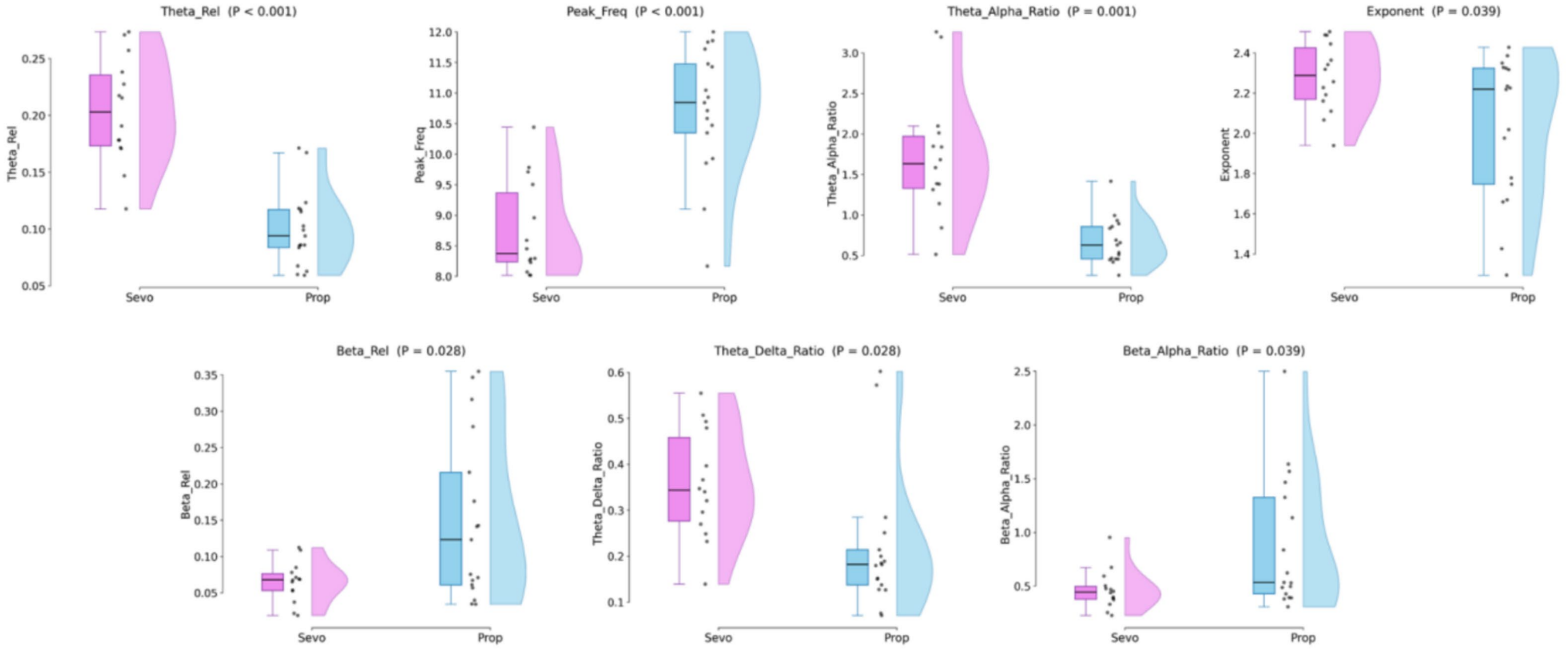
Distribution of key neurophysiological biomarkers. Raincloud plots illustrating the seven most statistically significant features (*p* < 0.05) identified by the multivariate framework. The plots compare Sevoflurane (pink) and Propofol (blue) cohorts. Each panel combines individual data points (jittered), boxplots (median and interquartile range), and kernel density estimates (half-violin) to visualize the data distribution.

The individual-level distributions of these key discriminatory features are visualized in Figure 5. As illustrated by the raincloud plots, the seven most statistically significant features (*p* < 0.05) distinctly stratify the neurophysiological regimes. The Sevoflurane epochs (pink) demonstrate a significantly broader distribution of power within the theta range, characterized by elevated Theta_Rel, Theta_Alpha_Ratio, and Theta_Delta_Ratio. In contrast, Propofol epochs (blue) are characterized by higher Beta_Rel and Beta_Alpha_Ratio, as well as a significantly higher alpha peak frequency (Peak_Freq), reflecting a stable, high-energy oscillation at a faster frequency tier.

Crucially, moving beyond periodic oscillations, the aperiodic Exponent is also visualized as a statistically significant differentiator (*p* = 0.039). The Sevoflurane group maintains a systematically steeper spectral slope (higher exponent), confirming that the background aperiodic dynamics provide critical and statistically robust contextual information for the random forest classifier, further solidifying its high ranking in the SHAP analysis.

## Discussion

### Distinct neurophysiological states of maintenance anesthesia

The results of this study indicate that although Propofol and Sevoflurane are both categorized as GABAergic anesthetics, the neurophysiological states associated with the maintenance of unconsciousness are fundamentally distinct. The capacity of the multivariate statistical framework to differentiate these regimens demonstrates that pharmacological effects are encoded across multiple specific spectral dimensions, supporting the transition from single univariate indices to multi-dimensional characterization (6, 7, 39). Building upon the foundational work of Abel et al. (23), the current analysis confirms that underlying cortical states are regimen-specific even at comparable surgical depths. The rigorous subject-level validation results and the spatial separability observed in the t-SNE analysis demonstrate that these drug-specific signatures are highly robust and identifiable across individuals. These findings indicate that resolving the dynamical regimes associated with specific molecular targets requires an integrated analysis of diverse structural neurophysiological markers (3, 5, 17).

### Theta dominance as the primary differentiator

The SHAP analysis revealed a profound dominance of theta-band dynamics in driving agent classification, with relative theta power (Theta_Rel) and theta-related ratios (Theta_Alpha_Ratio, Theta_Delta_Ratio) serving as the primary differentiators. The Sevoflurane group exhibited significantly higher relative theta power, a finding congruent with recent clinical evidence indicating that sevoflurane-maintained anesthesia is characterized by a specific low-frequency profile that diverges from pure intravenous GABAergic modulation (40).

Crucially, this theta dominance in the Sevoflurane group contradicts the expected effects of physiological aging. Typically, older adults exhibit attenuated spectral power in the slow-wave range (22). However, the Sevoflurane cohort, despite being significantly older than the Propofol cohort (50 vs. 32 years), demonstrated a paradoxical increase in theta power. This directionality suggests that the observed high-theta signature is a potent pharmacological effect of Sevoflurane that overrides the age-related attenuation. This interpretation is further substantiated by recent work from Guessous et al. (26), which established that theta-band power remains a robust signature of sevoflurane anesthesia even after statistical adjustment for age and dosage, a relationship absent in propofol protocols.

Mechanistically, this theta-predominant morphology may result from the polypharmacological profile of volatile agents, which, beyond GABA_A_ modulation, affect T-type calcium and hyperpolarization-activated cyclic nucleotide-gated channels, regulating thalamocortical oscillations in a manner distinct from propofol (3, 28, 41, 42).

### Distinct alpha oscillatory dynamics

The difference in alpha peak frequency further elucidates distinct thalamocortical dynamics under the two anesthetic agents. The Propofol group exhibited an alpha oscillation centered at approximately 10.88 Hz, whereas the Sevoflurane group demonstrated a consistent downshift to approximately 8.78 Hz (*p* < 0.001). While aging is known to contribute to a slowing of the alpha rhythm, the magnitude of this shift (*Δ* ≈ 2.1 Hz) closely aligns with recent findings from Dragovic et al. (43), who reported a comparable ~2 Hz difference between propofol (10.6 Hz) and sevoflurane (8.56 Hz) in an age-matched cohort. The fact that the alpha peak in the older Sevoflurane group (8.78 Hz) is nearly identical to their age-matched sevoflurane group (8.56 Hz) suggests that the observed downshift reflects a pharmacological effect of sevoflurane beyond what would be expected from age alone.

Crucially, while the alpha peak frequency was a top-tier discriminator, the width of the alpha peak (Alpha Bandwidth) showed no statistical difference between the regimens (*p* = 0.263) and ranked lowest in the multivariate SHAP analysis. This finding is perfectly corroborated by Dragovic et al. (43), who similarly reported no significant difference in peak width between these agents. Collectively, these results demonstrate that the rhythmic differentiation between intravenous and volatile GABAergic agents resides in the specific temporal scale (frequency) of thalamocortical loop integration, rather than the bandwidth of the generated oscillations (17, 26, 44, 45).

### The interplay of aperiodic dynamics and pharmacological signatures

The analysis of aperiodic (1/f) background activity revealed a significant divergence in global spectral architecture. The Sevoflurane group exhibited a steeper aperiodic exponent compared to the Propofol group (2.37 vs. 2.07, *p* = 0.039), an observation that highlights a critical interaction between aging and drug effects. Physiologically, advancing age is associated with a flattening of the power spectrum (decreased exponent) due to increased neural noise and asynchronous cortical activity (46). Consequently, the significantly older Sevoflurane cohort (mean 50 years) in this study would be expected to exhibit a noticeably flatter aperiodic slope than the younger Propofol cohort (mean 32 years).

However, spectral decomposition revealed the opposite trend: the older Sevoflurane group maintained a significantly steeper aperiodic decay. This apparent paradox aligns with recent spectral analyses demonstrating that flurane-based anesthesia intrinsically induces a steeper aperiodic slope compared to propofol (43, 47). The observation that the Sevoflurane cohort exhibits a higher exponent, despite the opposing physiological force of advanced age, indicates a potent, drug-specific steepening effect on background cortical dynamics. This pharmacological modulation effectively overrides the expected age-related spectral flattening, confirming that the steepened aperiodic slope serves as an intrinsic signature of the volatile anesthetic rather than a demographic artifact.

This finding is further supported by the multivariate model, which consistently identified the aperiodic exponent as a critical feature (SHAP Rank 5) for drug classification. As emphasized by Connor (2026), separating these aperiodic dynamics is essential for accurately distinguishing anesthetic agents (16). The robust performance of this framework demonstrates that the aperiodic component remains a vital, independent dimension of the combinatorial neural signature, serving as necessary contextual background for interpreting discrete oscillatory peaks.

### Signal complexity and spectral regularity

Complementing the spectral findings, the analysis of signal complexity revealed that univariate measures of stochasticity provided negligible discriminatory value. Specifically, Spectral Entropy (SpecEn) showed no statistical difference between the Propofol and Sevoflurane regimens (*p* = 0.721) and ranked near the bottom in the multivariate SHAP analysis. This lack of significance aligns with the framework established by Purdon and Brown, which emphasizes the analysis of drug-specific oscillatory patterns rather than aggregate complexity indices (1, 6).

While general anesthesia universally reduces overall EEG complexity and information integration compared to wakefulness (5, 30), the results indicate that the degree of spectral randomness remains comparable between Propofol and Sevoflurane during the steady-state maintenance phase. Consequently, the neurophysiological signatures that successfully distinguish these two GABAergic regimens are driven almost exclusively by structural shifts in oscillation frequency (e.g., alpha peak shift, theta prominence) and background power decay (aperiodic slope), rather than the overall disorder or complexity of the broad EEG signal.

### Clinical implications and limitations

The identification of divergent periodic and aperiodic signatures is relevant to precision anesthesia monitoring. As multimodal and opioid-free anesthesia (OFA) protocols become more prevalent, the use of monitoring tools that can resolve agent-specific neurophysiological effects is supported (3, 40). The observed theta-band prominence and the shift in alpha peak frequency under Sevoflurane reflect the distinct modulation of specific neural circuits. Integrating these specific periodic markers with aperiodic slope dynamics may facilitate earlier risk stratification, as variations in these parameters are correlated with neurocognitive vulnerability and postoperative delirium (18–20). The findings support a transition toward multidimensional monitoring protocols that incorporate drug-specific dynamics to enhance the precision of anesthetic management.

Several limitations of the present study warrant consideration. First, the open-access dataset provided demographic information only as group aggregates, precluding the application of individual-level covariate analysis (e.g., ANCOVA) to statistically adjust for the significant age disparity between the cohorts. Consequently, the contributions of physiological aging versus pharmacological modulation to the spectral features must be interpreted as a composite effect, although the directionality of the aperiodic exponent strongly suggests a dominant pharmacological driver. Second, the retrospective dataset did not provide detailed anesthetic concentration data (e.g., MAC or TCI targets), limiting the ability to standardize pharmacological depth. While the analysis was strictly confined to the steady-state maintenance phase to approximate functional equivalence, it cannot be ruled out that the observed spectral differences may partly reflect variations in cortical depression levels. Third, the lack of detailed medical histories regarding chronic medication use or pre-existing neurocognitive conditions poses a potential limitation, as unmeasured comorbidities such as mild cognitive impairment could influence quantitative EEG biomarkers (48). Finally, while the subject-level validation of the multivariate model demonstrated robust internal validity, future prospective studies utilizing age-matched cohorts and controlled anesthetic concentrations are necessary to confirm the generalizability of these neural signatures across diverse clinical populations.

## Conclusion

In conclusion, this comparative study identifies that Propofol and Sevoflurane anesthesia are characterized by divergent neurophysiological signatures during the steady-state maintenance phase. The differentiation of these regimens is primarily driven by robust, drug-specific shifts in periodic oscillatory frequencies, specifically, the prominence of theta-band power and a significant alpha peak downshift under Sevoflurane, alongside a distinct divergence in aperiodic background dynamics. Notably, the steeper aperiodic slope observed in the Sevoflurane group effectively overrides the expected physiological flattening associated with advancing age, demonstrating a potent and intrinsic pharmacological modulation. Conversely, features such as alpha bandwidth and aggregate signal complexity provided negligible discriminatory value. These findings support a transition toward multidimensional monitoring protocols that incorporate the specific structural features of both oscillatory and aperiodic dynamics to enhance the precision of individualized anesthetic management. Future prospective studies utilizing age-matched cohorts and controlled anesthetic concentrations are warranted to validate these signatures and further elucidate the complex interactions between aging and drug-specific neurophysiology.

## Data Availability

Publicly available datasets were analyzed in this study. This data can be found at: “GABAergic Anesthetic-Induced Unconsciousness and Recovery” https://github.com/johnabel/GABAergic_unconsciousness.
